# Supplementary material for: NKX2-5 Variant in Two Siblings with Thyroid Hemiagenesis
Source: Int J Mol Sci. 2022 Mar 21;23(6):3414. doi: 10.3390/ijms23063414 (PMC8950672; doi:10.3390/ijms23063414)
Supplement: Supplementary file 1 [file ijms-23-03414-s001.zip › ijms-1628137-supplementary.pdf]

Communication

NKX2-5 Variant in Two Siblings with Thyroid Hemiagenesis

Ewelina Szczepanek-Parulska [1,*], Bartłomiej Budny [1], Martyna Borowczyk [2], Igor Zhukov [3], Kosma Szutkowski [4], Katarzyna Zawadzka [5], Raiha Tahir [1], Andrzej Minczykowski [6], Marek Niedziela [7,†] and Marek Ruchała [1,†]

1 Department of Endocrinology, Metabolism and Internal Diseases, Poznan University of Medical Sciences, 61-701 Poznan, Poland; bbudny@ump.edu.pl (B.B.); raiha.tahir@outlook.com (R.T.); mruchala@ump.edu.pl (M.R.)
2 Department of Medical Simulation, Poznan University of Medical Sciences, 61-701 Poznan, Poland; martyna.borowczyk@ump.edu.pl
3 Polish Academy of Sciences, Institute of Biochemistry and Biophysics, 02-106 Warsaw, Poland; igor@ibb.waw.pl
4 NanoBioMedical Centre, Adam Mickiewicz University, 61-614 Poznan, Poland; kosma_sz@amu.edu.pl
5 MNM Diagnostics Sp. z o.o, 61-695 Poznan, Poland; katarzyna.zawadzka@mnm.bio
6 Department of Intensive Cardiological Care and Internal Medicine, Poznan University of Medical Sciences, 61-701 Poznan, Poland; anmin@ump.edu.pl
7 Department of Pediatric Endocrinology and Rheumatology, Institute of Pediatrics, Poznan University of Medical Sciences, 61-701 Poznan, Poland; mniedzie@ump.edu.pl
* Correspondence: ewelina@ump.edu.pl; Tel.: +48-61-869-13-30 (ext. 1682)
† These authors contributed equally to this work.

Supplementary Materials

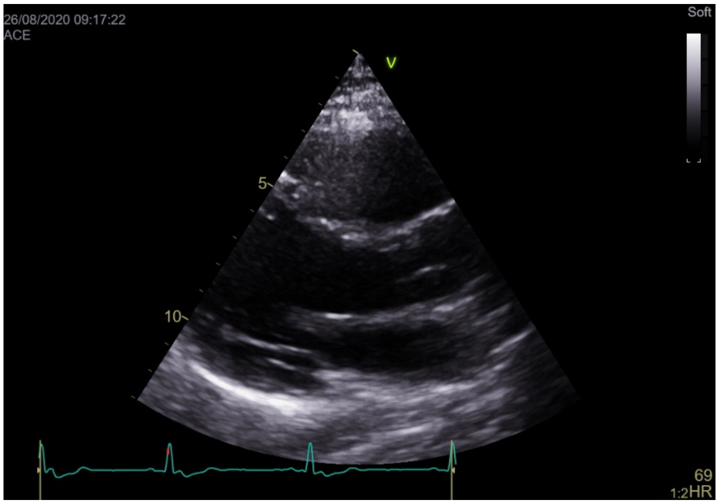

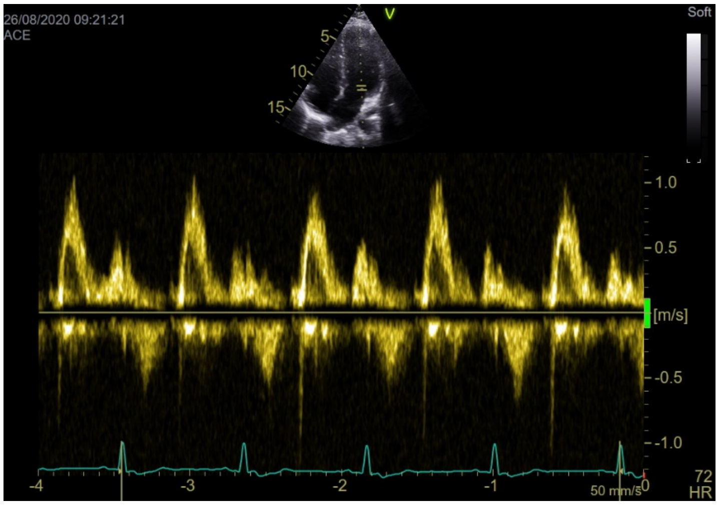
26/08/2020 09:21:21
ACE
Soft
72
HR
50 mm/s

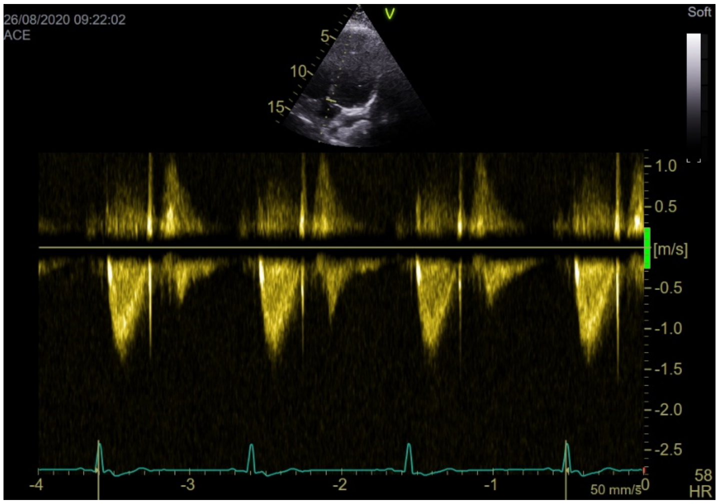
26/08/2020 09:22:02
ACE
Soft
58
HR
50 mm/s

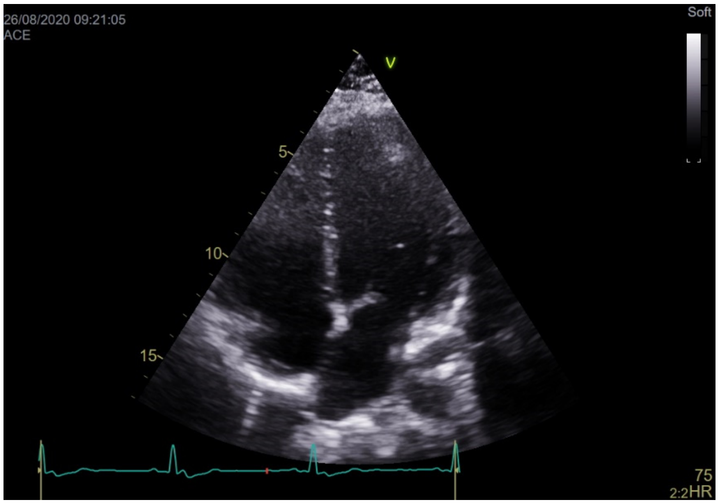
26/08/2020 09:21:05
ACE
Soft
75
2:2HR

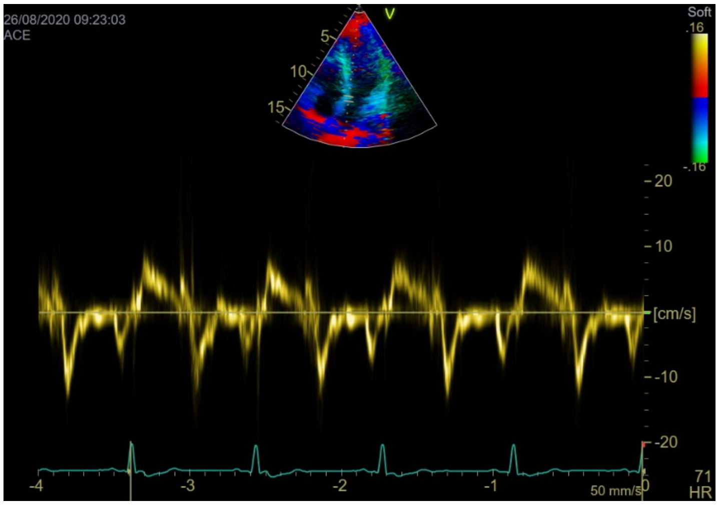

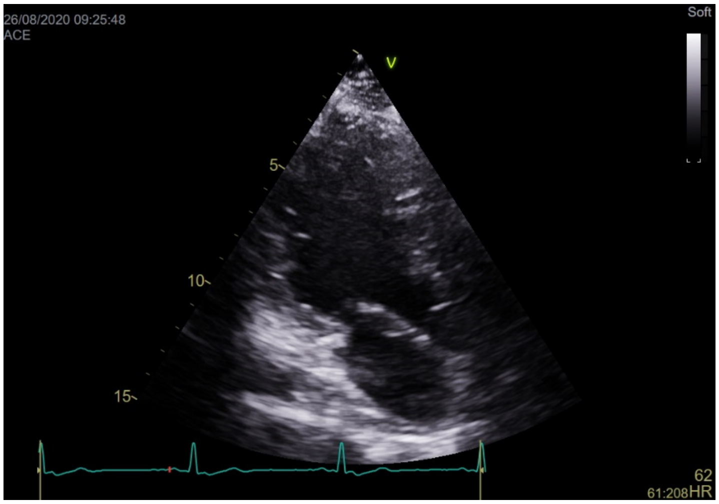

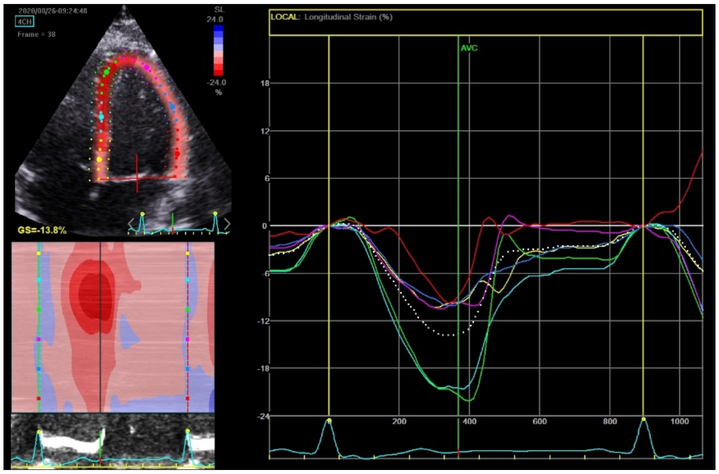

Supplementary Figures S1–S7 Images from echocardiographic examination of the male proband. 1 - parasternal long axis view (PLAX) view, 2D presentation, 2 - 4C view, pulsed wave doppler (PW), mitral inflow, 3 - 5C view, continuous wave doppler (CW), aortic valve flow, 4 - 4C view, 2D presentation, 5 - 4C view, tissue doppler (TDI), movement of the mitral valve ring, 6 - 3C view, 2D presentation, 7- 4C view, left ventricular strain.
