# Supplementary material for: NKX2-5 Variant in Two Siblings with Thyroid Hemiagenesis
Source: Int J Mol Sci. 2022 Mar 21;23(6):3414. doi: 10.3390/ijms23063414 (PMC8950672; doi:10.3390/ijms23063414)
Supplement: Supplementary file 1 [file ijms-23-03414-s001.zip › Suppl 10 Reported variants in NKX2-5 3D model.pdf]

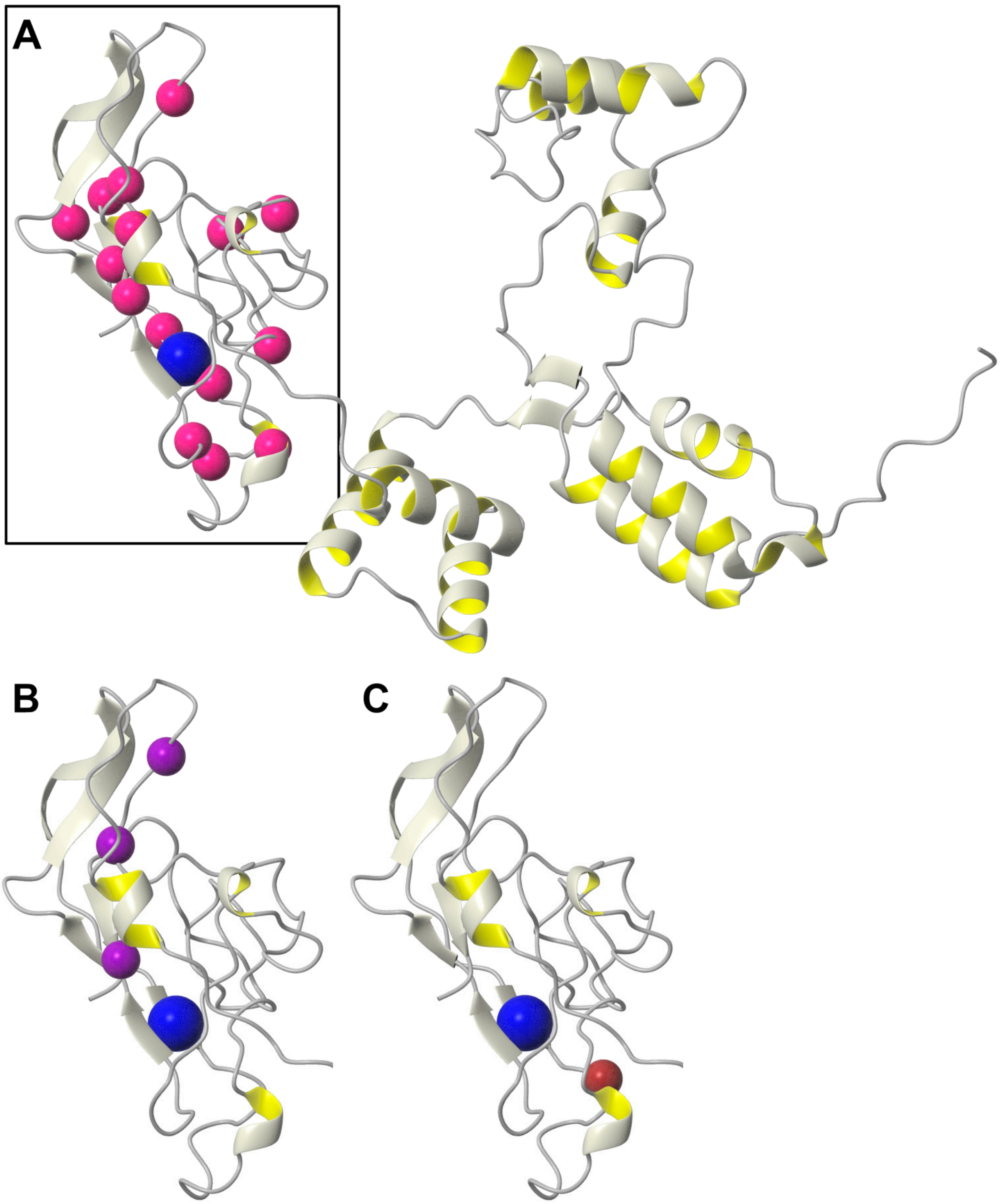

3D model of the model NKX2-5 p.P280L variant proposed on computer simulations. The mutation position of Leu280 is highlighted by blue ball. The pathogenic mutations described previously in literature and reported in Uniprot database (access number P52952) are presented. **A)** 15 mutations responsible for atrioventricular conduction defects (ASD7). Mutation positions are shown as red balls. **B)** Three pathological mutant at positions 216, 219, and 323 (violet balls) responsible for Tetralogy of Fallot (TOF). **C)** One pathological mutant in position 283, responsible for Ventricular septal defect 3 (VSD3).
