# Supplementary material for: NKX2-5 Variant in Two Siblings with Thyroid Hemiagenesis
Source: Int J Mol Sci. 2022 Mar 21;23(6):3414. doi: 10.3390/ijms23063414 (PMC8950672; doi:10.3390/ijms23063414)
Supplement: Supplementary file 1 [file ijms-23-03414-s001.zip › Suppl 9 Modelling data/Supplementary 9.pdf]

The homology modelling results

1. PSI-BLAST

3 PSI-BLAST iterations were performed to extract the position specific scoring matrix (PSSM) from UniRef90 database. 109 hits were found in overall. 5 templates were selected with a highest total scores for both proteins. Templates for homology modelling for both NKX2-5 normal and variant are shown in Tab.S1.

Table S1. The selected templates for homology modelling (both normal and variant)

Template	Total score	BLAST E-value	Align score	Cover	ID	Resolution	Header
1	90.95	2,00E-21	240.0	53%	5FLV-I	3.00 A	XRAY Homeobox protein Nkx-2.5 [Mus musculus] <NKX25_MOUSE(134-197)> (226 residues with quality score 0.717), released 2016-02-10
2	43.08	2,00E-26	255.0	21%	1FTT-A	Unknown or NMR	NMR Homeobox protein Nkx-2.1 [Rattus norvegicus] <NKX21_RAT(161-227)> (68 residues with quality score 0.805), released 1996-01-29
3	35.92	2,00E-18	113.0	32%	6M3D-C	1.60 A	XRAY Segmentation polarity homeobox protein engrailed [Drosophila melanogaster] <HMEN_DROME(453-512)> (108 residues with quality score 1.000), released 2020-09-16
4	30.41	4,00E-20	175.0	21%	2L9R-A	Unknown or NMR	NMR Homeobox protein Nkx-3.1 [Homo sapiens] <NKX31_HUMAN(132-189)> (69 residues with quality score 0.816), released 2011-04-20
5	30.41	2,00E-25	164.0	19%	4RDU-D	1.85 A	XRAY Homeobox protein DLX-5 [Homo sapiens] <DLX5_HUMAN(135-198)> (62 residues with quality score 0.969), released 2014-11-19

2. FASTA sequences

Fasta sequence for NKX2-5 (normal)

>NKX2-5

MFPSPALTPTPFSVKDILNLEQQQRSLAAAGELSARLEATLAPSSCMLAAFKPEAYAGPE

AAAPGLPELRAELGRAPSPAKCASAFPAAPAFYPRAYSDPDPAKDPRAEKKELCALQKAV

ELEKTEADNAERPRARRRRKPRVLFSQAQVYELERRFKQQRYLSAPERDQLASVLKLTST

QVKIWFQNRRYKCKRQRQDQTLELVGLPPPPPPPARRIAVPVLVRDGKPCLGDSAPYAPA

YGVGLNPYGYNAYPAYPGYGGAACSPGYSCTAAYPAGPSPAQPATAAANNNFVNFGVGDL

NAVQSPGIPQSNSGVSTLHGIRAW

Fasta sequence for NKX2-5 (mutation)

>NKX2-5 P.PRO280LEU.

MFPSPALTPTPFSVKDILNLEQQQRSLAAAGELSARLEATLAPSSCMLAAFKPEAYAGPE

AAAPGLPELRAELGRAPSPAKCASAFPAAPAFYPRAYSDPDPAKDPRAEKKELCALQKAV

ELEKTEADNAERPRARRRRKPRVLFSQAQVYELERRFKQQRYLSAPERDQLASVLKLTST

QVKIWFQNRRYKCKRQRQDQTLELVGLPPPPPPPARRIAVPVLVRDGKPCLGDSAPYAPA

YGVGLNPYGYNAYPAYPGYGGAACSPGYSCTAAYPAGPSLAQPATAAANNNFVNFGVGDL

NAVQSPGIPQSNSGVSTLHGIRAW

3. Models used during homology modelling for NKX2-5 normal

Table S2. The list of 13 models sorted by their Z-scores for NKX2-5 normal

Rank	Z-score	Structure	State	Model ID	Filename	Original number	Residues	Comment
1	1.443	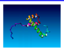	monomer	1FTT-A	nkx2-5_1ftt-a.yob	6	143-197	Optimal
2	1.165	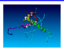	monomer	4RDU-D	nkx2-5_4rdu-d.yob	13	138-198	Optimal
3	0.799	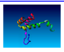	monomer	2L9R-A	nkx2-5_2l9r-a.yob	12	145-196	Optimal
4	-0.805	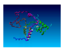	monomer	6M3D-C01	nkx2-5_6m3d-c01.yob	7	9-197	Good
5	-0.894	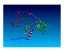	monomer	6M3D-C03	nkx2-5_6m3d-c03.yob	9	9-197	Good
6	-0.978	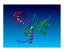	monomer	6M3D-C05	nkx2-5_6m3d-c05.yob	11	9-197	Good
7	-1.177	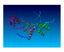	monomer	6M3D-C04	nkx2-5_6m3d-c04.yob	10	9-197	Satisfactory
8	-1.296	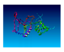	monomer	6M3D-C02	nkx2-5_6m3d-c02.yob	8	9-197	Satisfactory
9	-2.466	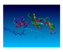	monomer	5FLV-I02	nkx2-5_5flv-i02.yob	2	141-324	Poor
10	-2.606	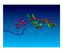	monomer	5FLV-I01	nkx2-5_5flv-i01.yob	1	141-324	Poor
11	-2.684	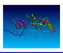	monomer	5FLV-I03	nkx2-5_5flv-i03.yob	3	141-324	Poor
12	-2.859	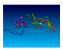	monomer	5FLV-I04	nkx2-5_5flv-i04.yob	4	141-324	Poor
13	-2.936	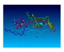	monomer	5FLV-I05	nkx2-5_5flv-i05.yob	5	141-324	Poor

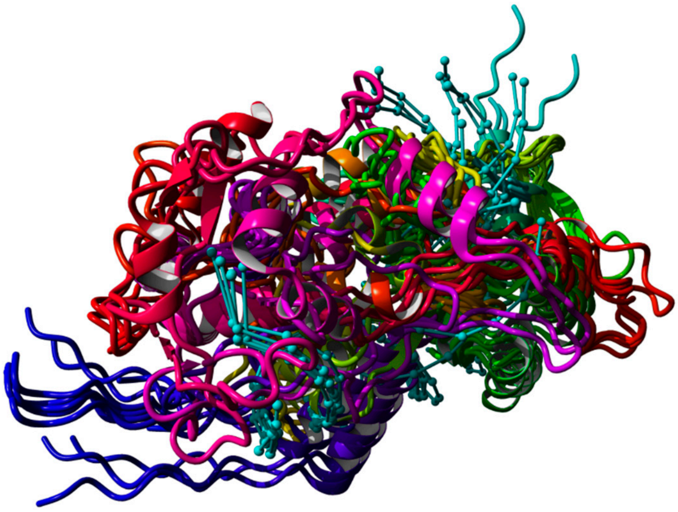

Figure S8. Aligned models from Tab. S2 (file nkx2-5_normal_13models.pdb)

Table S3. List of hybrid models obtained from the best parts of models listed in Tab.S2

Transfer	First residue	Last residue	Length	From model	Score
1	126	210	85	4RDU-D	**-6.419**
2	1	145	145	6M3D-C05	**-4.417** accepted
3	190	324	135	5FLV-I03	**-2.657** accepted
6	144	157	14	6M3D-C02	**-2.655** accepted
7	119	127	9	6M3D-C03	**-2.638** accepted
8	141	157	17	6M3D-C04	**-2.604** accepted
9	140	159	20	6M3D-C04	**-2.597** accepted
10	146	159	14	6M3D-C04	**-2.578** accepted
11	149	157	9	6M3D-C02	**-2.573** accepted
12	146	157	12	6M3D-C02	**-2.540** accepted
13	148	159	12	6M3D-C04	**-2.535** accepted
16	210	215	6	5FLV-I02	**-2.530** accepted

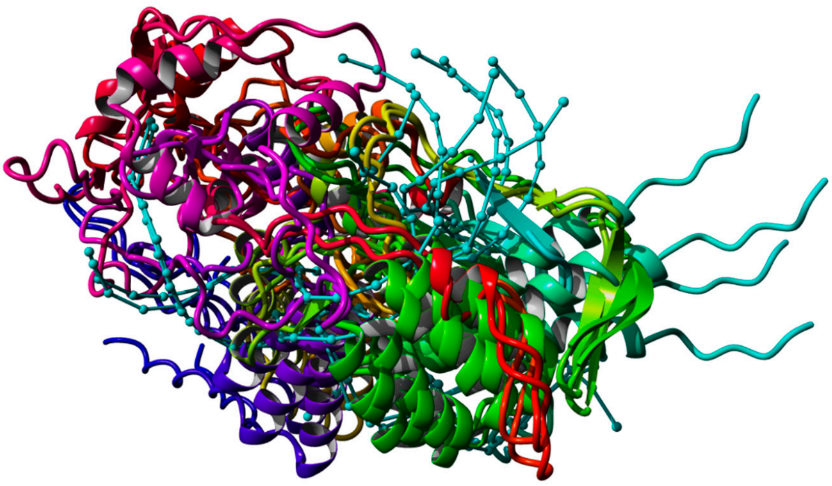

Figure S9 Superimposed hybrid models from Tab.S3 (file nkx2-5_normal_7models.pdb)

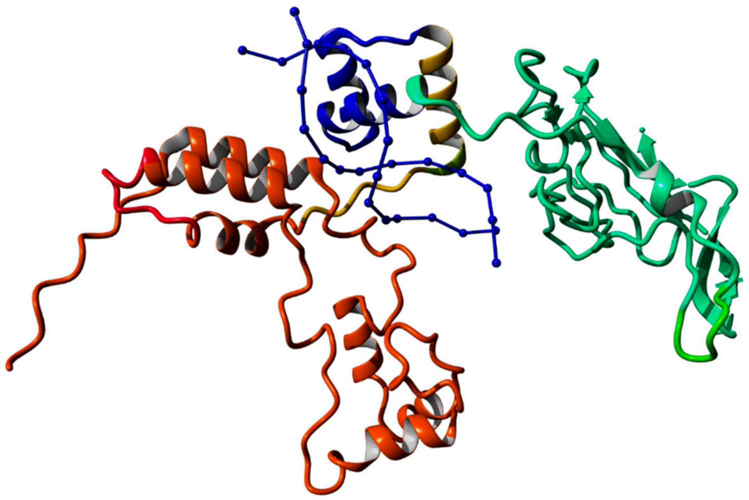

Figure S10 Final model for NKX2-5 normal with an overall Z-score -2.250 (Dihedrals -3.003 bad, Packing 1D -1.805 satisfactory, Packing 3D -2.389 poor). (file nkx2-5_normal.pdb)

4. Models used during homology modelling for NKX2-5 variant

Table S4. The list of 13 models sorted by their Z-scores for NKX2-5 variant

Rank	Z-score	Structure	State	Model ID	Filename	Original number	Residues	Comment
1	1.443	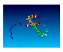	monomer	1FTT-A	nkx2-5_p.Pro280Leu._1ftt-a.yob	6	143-197	Optimal
2	1.165	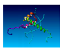	monomer	4RDU-D	nkx2-5_p.Pro280Leu._4rdu-d.yob	13	138-198	Optimal
3	0.799	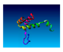	monomer	2L9R-A	nkx2-5_p.Pro280Leu._2l9r-a.yob	12	145-196	Optimal
4	-0.805	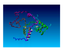	monomer	6M3D-C01	nkx2-5_p.Pro280Leu._6m3d-c01.yob	7	9-197	Good
5	-0.894	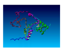	monomer	6M3D-C03	nkx2-5_p.Pro280Leu._6m3d-c03.yob	9	9-197	Good
6	-0.978	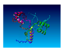	monomer	6M3D-C05	nkx2-5_p.Pro280Leu._6m3d-c05.yob	11	9-197	Good
7	-1.177	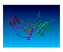	monomer	6M3D-C04	nkx2-5_p.Pro280Leu._6m3d-c04.yob	10	9-197	Satisfactory
8	-1.296	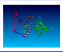	monomer	6M3D-C02	nkx2-5_p.Pro280Leu._6m3d-c02.yob	8	9-197	Satisfactory
9	-2.359	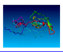	monomer	5FLV-I02	nkx2-5_p.Pro280Leu._5flv-i02.yob	2	141-324	Poor
10	-2.492	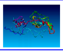	monomer	5FLV-I04	nkx2-5_p.Pro280Leu._5flv-i04.yob	4	141-324	Poor
11	-2.774	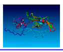	monomer	5FLV-I05	nkx2-5_p.Pro280Leu._5flv-i05.yob	5	141-324	Poor
12	-2.828	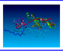	monomer	5FLV-I01	nkx2-5_p.Pro280Leu._5flv-i01.yob	1	141-324	Poor
13	-2.973	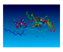	monomer	5FLV-I03	nkx2-5_p.Pro280Leu._5flv-i03.yob	3	141-324	Poor

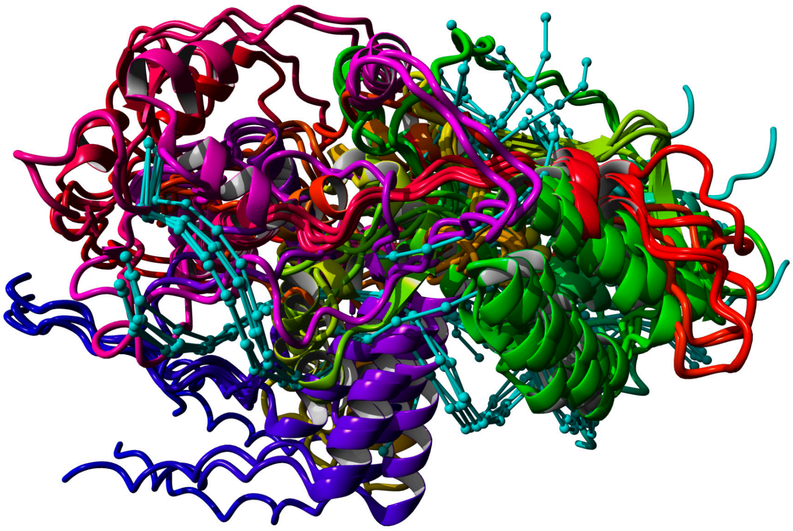

Figure S11. Aligned models from Tab. S2 (file nkx2-5_variant_13models.pdb)

Table S5. List of hybrid models obtained from the best parts of models listed in Tab.S4

Transfer	First residue	Last residue	Length	From model	Score
1	126	210	85	4RDU-D	**-6.419**
2	1	145	145	6M3D-C05	**-4.419** accepted
3	190	324	135	5FLV-I05	**-2.704** accepted
4	141	157	17	6M3D-C04	**-2.688** accepted
5	146	159	14	6M3D-C04	**-2.678** accepted
6	119	127	9	6M3D-C03	**-2.649** accepted
7	144	157	14	6M3D-C02	**-2.629** accepted
8	152	157	6	6M3D-C04	**-2.612** accepted
10	181	198	18	5FLV-I02	**-2.608** accepted
11	170	187	18	5FLV-I02	**-2.574** accepted
12	119	122	4	6M3D-C03	**-2.572** accepted
13	184	189	6	6M3D-C02	**-2.549** accepted
15	210	215	6	5FLV-I02	**-2.545** accepted
16	119	122	4	6M3D-C03	**-2.522** accepted
18	152	155	4	6M3D-C04	**-2.514** accepted
20	209	214	6	5FLV-I05	**-2.511** accepted

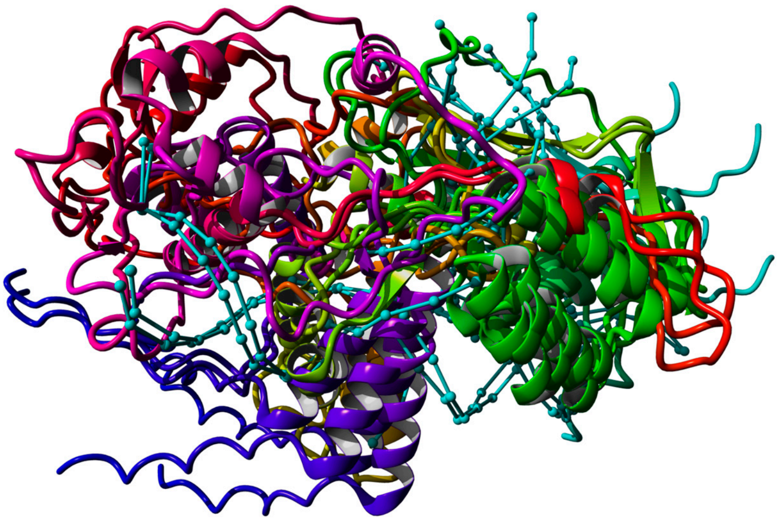

Figure S12. Aligned models from Tab. S5 (file nkx2-5_variant_7models.pdb)

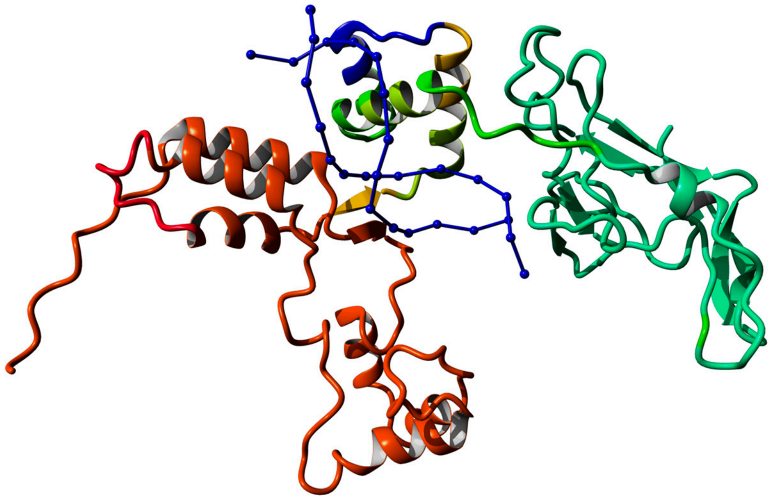

Figure S13. Final model for NKX2-5 variant with an overall Z-score -2.310 (Dihedrals -3.261 bad, Packing 1D - 1.845 satisfactory, Packing 3D -2.404 poor). (file nkx2-5_variant.pdb)
